# The impact of early enteral nutrition on 28-day mortality in septic shock: a cohort study

**DOI:** 10.3389/fnut.2026.1789277

**Published:** 2026-05-11

**Authors:** Min Mo, Jianfeng Xie, Changde Wu, Ling Liu, Hui Chen, Yi Yang

**Affiliations:** 1 Jiangsu Provincial Key Laboratory of Critical Care Medicine, Department of Critical Care Medicine, Zhongda Hospital, School of Medicine, Southeast University, Nanjing, China; 2 Department of Critical Care Medicine, Nanjing Lishui People's Hospital, Zhongda Hospital Lishui Branch, Southeast University, Nanjing, China

**Keywords:** early enteral nutrition, mortality, sepsis, septic shock, vasopressor dose

## Abstract

**Background:**

Enteral nutrition (EN) in septic shock patients is a double-edged sword. The impact of early enteral nutrition (EEN) on mortality in septic shock patients remains uncertain, and whether this association is modified by vasopressor dosage is also unclear.

**Objective:**

The study aimed to investigate the association between EEN and 28-day in-hospital mortality in patients with septic shock and to explore whether the association varied across different dosages of vasopressors during the first 24 h.

**Methods:**

Data were extracted from the Chinese Database in Intensive Care (CDIC). Adult septic patients who received vasopressors within 24 h of intensive care unit (ICU) admission were eligible for inclusion. The primary exposure was whether patients received early nutrition within the first 24 h after ICU admission (the EEN group). The primary outcome was 28-day mortality. Multivariate Cox regression models were employed to assess the association between the EEN group and 28-day mortality in the overall population and in patients receiving different vasopressor dosing intensity (VDI) levels. VDI was defined as the total vasopressor dose expressed in norepinephrine equivalents (NEEs), calculated as the time-weighted average over the first 24 h.

**Results:**

A total of 26,478 patients were screened and, ultimately, 1855 patients met the inclusion criteria. The median Sequential Organ Failure Assessment (SOFA) score was 11 [interquartile range (IQR): 8–14], and the Acute Physiology and Chronic Health Evaluation (APACHE) II score was 20 (IQR: 15–26). The 28-day in-hospital mortality rate was 19.7%. Compared to patients in the EEN group, those in the late enteral nutrition (LEN) group required higher vasopressor doses (8.7 vs. 10.6 ug/min, *p*<0.001) and had a higher 28-day mortality rate (16.6% vs. 21.1%, *p* = 0.03). After adjusting for confounding factors, EEN was not significantly associated with a reduction in the risk of 28-day mortality [Hazard ratio (HR) 0.832, 95% confidence interval (CI) (0.654–1.059), *p* = 0.135]. When stratified by VDI (norepinephrine equivalents), EEN exhibited a trend toward reduced 28-day mortality; however, this difference failed to reach statistical significance in patients with VDI < 15ug/min [HR 0.738, 95% CI (0.523–1.042), *p* = 0.084]. In contrast, EEN had no discernible impact on 28-day mortality risk in patients with VDI ≥ 15ug/min [HR 0.926, 95% CI (0.660–1.298, *p* = 0.655)].

**Conclusion:**

Early enteral nutrition was not associated with a lower 28-day mortality risk in patients with septic shock, and it only demonstrated a trend in patients receiving low doses of vasopressors.

## 1 Introduction

The adult guidelines for nutrition support in critical illness emphasize early enteral nutrition (EEN) as a crucial strategy for improving clinical outcomes (1), primarily because it promotes and maintains gastrointestinal mucosal integrity and function (2). The benefits of EEN include reduced infection rates and improved healing during intensive care unit (ICU) dependency, contributing to better short- and long-term outcomes (2–4), including reduced ICU and hospital stays. Although extensive randomized controlled trials and meta-analyses have confirmed that EEN positively impacts clinical outcomes and reduces costs (5–8), the effects of EEN in patients with septic shock are less well-established, with research presenting mixed results (9).

A large observational study demonstrated that in patients with stable hemodynamics receiving fluid resuscitation and vasopressors, EEN was associated with lower mortality rates compared to late enteral nutrition (LEN) (10). In addition, several non-randomized studies have indicated an improved survival rate among mechanically ventilated ICU patients with septic shock who received EEN (11–16). Conversely, a previous randomized clinical trial found that EEN did not reduce mortality or the risk of secondary infections. Notably, it was associated with a higher incidence of digestive complications when compared to early parenteral nutrition in patients receiving high-dose noradrenaline (median, 0.56 μg/kg/min) for septic shock (9). According to expert consensus, the Society of Critical Care Medicine (SCCM) and the American Society for Parenteral and Enteral Nutrition (ASPEN) 2016 Critical Care Nutrition Guidelines recommend cautiously initiating enteral nutrition (EN) in ICU patients with stable conditions (17). The severity of illness, particularly the dosage of vasopressors, may help explain the varying outcomes associated with EEN in patients with septic shock.

We hypothesized that the clinical effect of EEN may vary among patients with shock depending on the dose of norepinephrine received. We defined vasopressor dosing intensity (VDI) as the total dose of all vasopressors administered, expressed in norepinephrine equivalents (NEEs). Using this definition, our objective is to assess the effect of EEN on 28-day in-hospital mortality among patients with septic shock. In addition, we examined whether the effect of EEN differs across varying levels of vasopressor dosing intensity during the initial 24 h of treatment.

## 2 Materials and methods

### 2.1 Study design

The present study was a retrospective observational study, with data extracted from the Chinese Database in Intensive Care (CDIC). The CDIC includes data from 26,478 patients admitted to the Department of Critical Care Medicine, Zhongda Hospital, Southeast University, China, from January 2014 to March 2025. Details of the CDIC are provided in Additional file 1. The present study was approved by the Research Ethics Commission of Zhongda Hospital, Southeast University (2022ZDSYLL177-P01). The requirement for informed consent was waived.

### 2.2 Study participants

Adult septic patients who received vasopressors within 24 h of ICU admission were eligible for inclusion in this study. The diagnosis of sepsis was based on the Third International Consensus Definitions for Sepsis and Septic Shock (Sepsis-3) (18). Patients were excluded if they (i) died within 48 h after ICU admission; (ii) were admitted for gastrointestinal tract surgery, active gastrointestinal bleeding, ileus/bowel obstruction, bowel ischemia, or other contraindications to EN; (iii) were pregnant; or (iv) had missing data. For patients who were admitted to the ICU more than once, only the first ICU stay was included for analysis. Patients were subsequently divided into two groups according to the timing of EN: Those who started enteral nutrition within 24 h of admission were assigned to the EEN group, while the remainder were classified as the LEN group.

### 2.3 Data collection

Data from the CDIC representing baseline characteristics within the first 24 h after ICU admission were collected, including age, sex, body mass index (BMI) (kg/m^2^), admission type, comorbidities, and site of infection. The use of mechanical ventilation, renal replacement therapy (RRT), and vasopressors within 24 h after ICU admission was also extracted. Vasopressor administration was recorded as the maximum and minimum infusion doses administered during each 6-h period, and vasopressor dosing intensity (VDI) was calculated as the time-weighted average of NEE doses over 24 h. For each interval (0–3, 3–6, 6–12, and 12–24 h), the mean of the lowest and highest infusion rates for each vasopressor was converted to NEEs using the following formula: norepinephrine + dopamine/2 + epinephrine + phenylephrine/10. Detailed information is provided in the Supplementary material. Vital signs and laboratory variables were measured during the first 24 h in the ICU. The severity of illness was measured using the sequential organ failure assessment (SOFA) score and Acute Physiology and Chronic Health Evaluation II (APACHE II) score. If a variable was recorded more than once in the first 24 h, we used the value related to the greatest severity of sepsis.

### 2.4 Primary exposure and outcomes

The primary exposure was whether the patients received EN within the first 24 h after ICU admission (the EEN group). The primary outcome was 28-day mortality. The secondary outcomes included nosocomial pneumonia, length of hospital stay, and duration of mechanical ventilation.

### 2.5 Statistical analysis

Values were presented as mean (standard deviation) or median [interquartile range (IQR)] for continuous variables, as appropriate, and as counts and percentages for categorical variables. Comparisons between the groups were performed using the *X*^2^ test or Fisher’s exact test for categorical variables and Student’s *t-*test or the Mann–Whitney U test for continuous variables, as appropriate. Missing data for covariates (<20%) were imputed using the median for continuous variables and the mode for categorical variables. A *p*-value of < 0.05 was considered statistically significant when comparing differences. All statistical analyses were performed using R (version 4.1.3).

We employed a multivariate logistic regression model to explore the association between the timing of EN and 28-day mortality. Covariates were selected based on clinical relevance and established associations with mortality in critically ill patients, as identified in previous studies. These variables, including age, sex, BMI, VDI, APACHE II score, SOFA score, and lactate, were entered into the model as potential confounders. We then compared 28-day mortality between patients in the EEN and LEN groups using Kaplan–Meier curves and the log-rank test.

## 3 Results

**Figure 1:**
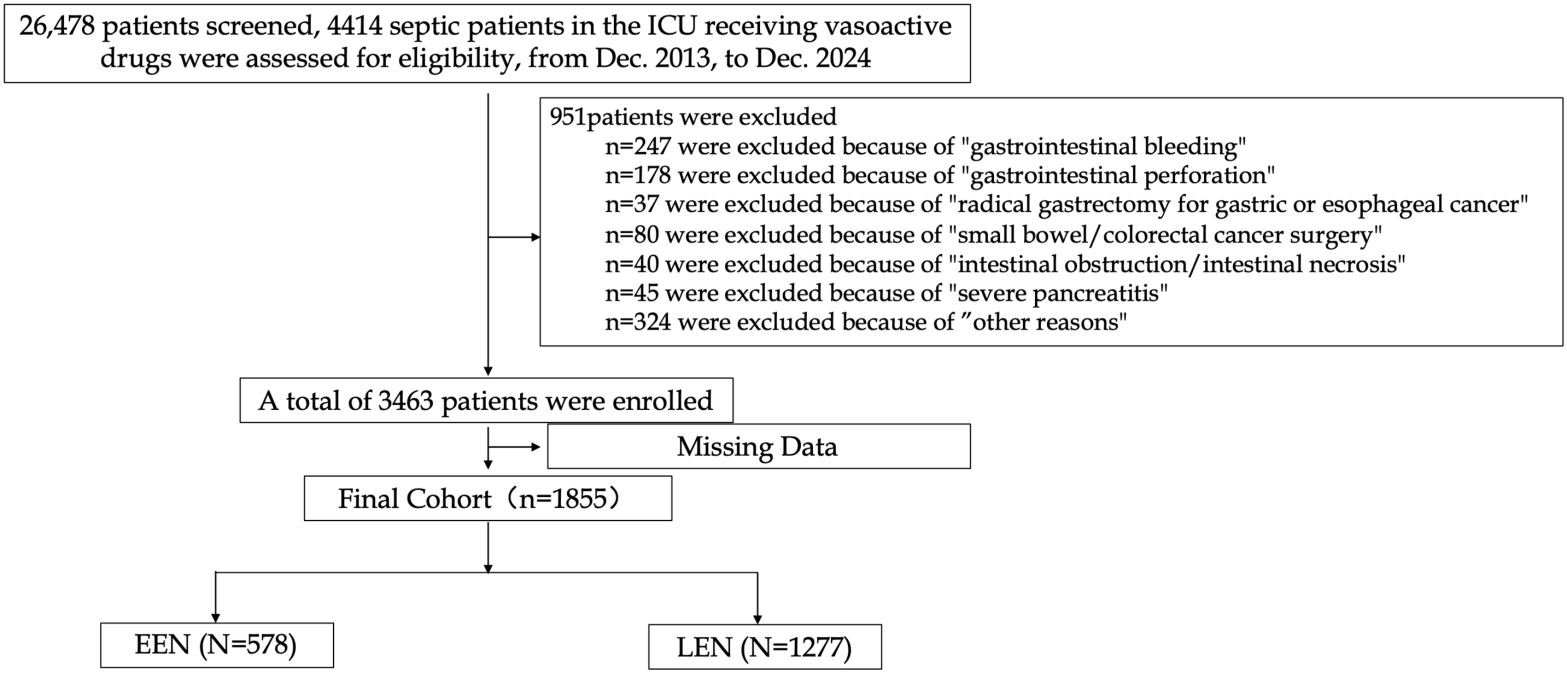
Flowchart of patient selection. ICU, intensive care unit; EEN, early enteral nutrition; LEN, late enteral nutrition.

A total of 26,478 patients from the CDIC database were initially screened, and 1,855 patients met the inclusion and exclusion criteria and were included in the final analysis (Figure 1). The median SOFA score was 11 (IQR: 8–14), and the median APACHE II score was 20 (IQR: 15–26). The 28-day all-cause mortality rate was 19.7%. Norepinephrine was the most frequently used vasopressor. The median vasopressor dosing intensity during the first 24 h was 10 μg/min norepinephrine equivalents (5–23.2 μg/min norepinephrine equivalents). Detailed baseline characteristics of the patient are presented in Table 1.

**Table 1:** Baseline characteristics of patients stratified by the timing of EN.

Variable	Total (*n* = 1855)	EEN (*n* = 578)	LEN (*n* = 1,277)	*p*-value
Age, median (IQR), years	68 (54, 77)	70 (56, 79)	66 (53, 76)	< 0.001
Sex (Male), *n* (%)	1,174 (63.3)	396 (68.5)	778 (60.9)	0.002
BMI, median (IQR), kg/m^2^	23.4 (20.8, 25.4)	22.9 (20.8, 25.4)	23.4 (20.8, 25.4)	0.177
APACHE II score, median (IQR)	20 (15, 26)	21 (16, 26)	19 (14, 26)	0.049
SOFA score, median (IQR)	11 (8, 14)	11 (8, 13)	11 (8, 14)	0.935
Infection site, *n* (%)				< 0.001
Pneumonia	1,150 (62)	429 (74.2)	721 (56.5)	
Abdominal	331 (17.8)	72 (12.5)	259 (20.3)	
Urinary tract	86 (4.6)	19 (3.3)	67 (5.2)	
Bloodstream infection	62 (3.3)	14 (2.4)	48 (3.8)	
Other	226 (12.2)	44 (7.6)	182 (14.3)	
Comorbidities, *n* (%)				
Hypertension	954 (51.4)	314 (54.3)	640 (50.1)	0.103
Diabetics	464 (25)	160 (27.7)	304 (23.8)	0.084
Congestive heart failure	407 (21.9)	119 (20.6)	288 (22.6)	0.375
Liver cirrhosis	33 (1.8)	8 (1.4)	25 (2)	0.499
Chronic kidney disease	115 (6.2)	31 (5.4)	84 (6.6)	0.368
Use of IMV, *n* (%)	1,418 (76.4)	489 (84.6)	929 (72.7)	< 0.001
Use of CRRT, *n* (%)	297 (16)	62 (10.7)	235 (18.4)	< 0.001
Fluid balance 24 h, mL	358 (−496, 1,386)	398 (−391, 1,293)	342 (−584, 1,452)	0.625
Volume of EN at 24 h, mL	0 (0, 500)	500 (500, 1,000)	0 (0, 0)	< 0.001
VDI, ug/min	10 (5, 23.2)	8.7 (5, 16.4)	10.6 (5, 26.9)	< 0.001
Vital signs				
Heart rate, bpm	102 (69, 121)	99 (68, 120)	105 (69, 122)	0.034
MAP, mm Hg	78 (67, 91)	81 (69, 92)	77 (66, 90)	< 0.001
Respiratory rate, bpm	22 (15, 28)	22 (15, 27)	22 (15, 28)	0.371
Temperature, °C	36.9 (36.2, 37.9)	37 (36.3, 37.9)	36.9 (36.2, 37.8)	0.208
Laboratory values				
Platelet, *10^9^/L	134 (86, 192)	153 (103.5, 209)	126 (80, 187)	< 0.001
pH	7.35 (7.29, 7.42)	7.38 (7.31, 7.44)	7.33 (7.28, 7.42)	< 0.001
PaO_2_, mmHg	134 (95, 179)	128 (91, 1712)	137 (97, 184)	0.083
Total bilirubin, μmol/L	16.9 (10.5, 28.2)	13.6 (9.2, 23.92)	17.8 (11.1, 30.25)	< 0.001
Serum creatinine, μmol/L	92 (63, 162.25)	87 (59, 138.5)	97 (66, 177)	< 0.001
Lactate, mmol/L	1.2 (0.8, 1.9)	1.1 (0.8, 1.5)	1.3 (0.9, 2.1)	< 0.001
C-reactive protein, mg/L	75.82 (24.5, 145)	73.75 (25.78, 140)	76.7 (23.6, 149)	0.991
Procalcitonin, ng/mL	2.06 (0.51, 9.2)	1.22 (0.33, 5.16)	2.57 (0.6, 10.64)	< 0.001
Outcomes				
ARDS, *n* (%)	895 (48.2)	276 (47.8)	619 (48.5)	0.812
AKI, *n* (%)	303 (16.3)	88 (15.2)	215 (16.8)	0.423
Hospital length of stay, median (IQR), days	17.57 (9.23, 28.65)	19.94 (11.02, 32.08)	16.58 (8.67, 26.81)	< 0.001
ICU length of stay, median (IQR), days	7.08 (2.9, 13.82)	10.53 (5.79, 17.83)	5.62 (2.28, 11.72)	< 0.001
28-day mortality, *n* (%)	365 (19.7)	96 (16.6)	269 (21.1)	0.03
ICU mortality, *n* (%)	242 (13)	70 (12.1)	172 (13.5)	0.465
Hospital mortality, *n* (%)	299 (16.1)	95 (16.4)	204 (16)	0.856

EEN, early enteral nutrition; LEN, late enteral nutrition; BMI, body mass index; APACHE II, Acute Physiology and Chronic Health Evaluation II score; SOFA, Sequential Organ Failure Assessment; IMV, invasive mechanical ventilation; CRRT, continuous renal replacement therapy; EN, enteral nutrition; VDI, vasopressor dosing intensity; MAP, mean arterial pressure; ARDS, acute respiratory distress syndrome; AKI, acute kidney injury; ICU, intensive care unit.

Among the 1,855 patients included in the study, 578 (31.2%) received EEN and 1,277 (68.8%) received late enteral nutrition (LEN). Patients in the EEN group were older (median age, 70 years vs. 66 years, *p* < 0.001) and had higher APACHE II scores (median, 21 vs. 19, *p* = 0.049) compared to those in the LEN group (Table 1). There were no significant differences between the two groups in the SOFA score [median 11 (IQR, 8–13) in the EEN group vs. 11 (IQR, 8–14) in the LEN group; *p* = 0.935]. There were also no differences in the incidence of ARDS and AKI between the groups (*p* = 0.812, *p* = 0.423). Patients in the EEN group had lower VDI (median 8.7 μg/min vs. 10.6 μg/min, *p*<0.001). Comorbidities were similar between the two groups.

**Figure 2:**
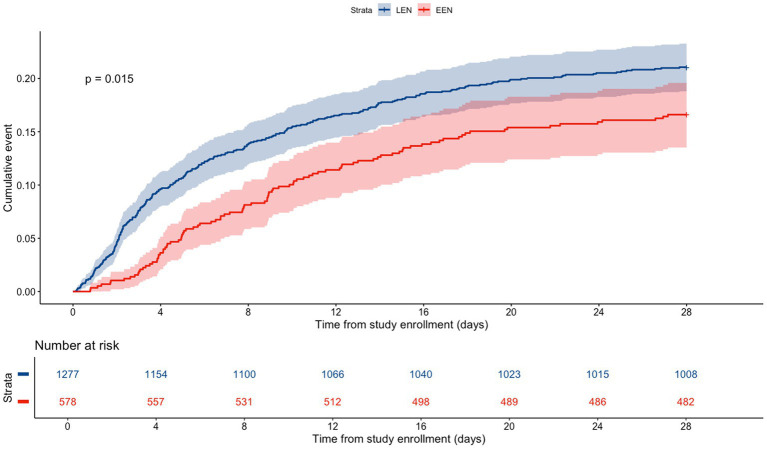
Kaplan–Meier estimates of cumulative incidence in septic patients, comparing the EEN and LEN groups. EEN, early enteral nutrition; LEN, late enteral nutrition.

The 28-day mortality rate was 16.6% in the EEN cohort and 21.1% in the LEN cohort (*p* = 0.03; Table 1). After adjusting for age, sex, BMI, VDI, APACHE II score, SOFA score, and lactate levels, Cox regression analysis revealed no significant difference in 28-day mortality. EEN was not associated with a reduced risk of mortality (Hazard ratio (HR) 0.832, 95% confidence interval (CI) (0.654–1.059), *p* = 0.135; Table 2; Figure 2).

**Table 2:** Association between EEN and 28-day mortality using the Cox proportional hazards regression model.

Variable	HR (95% CI)	*p*-value
EEN	0.832 (0.654–1.059)	0.135
Age	1.007 (0.999–1.013)	0.063
Male	1.069 (0.860–1.329)	0.546
BMI	0.962 (0.936–0.990)	0.008
VDI	1.008 (1.004–1.011)	<0.001
APACHE II	1.075 (1.058–1.092)	<0.001
SOFA score	1.013 (0.980–1.047)	0.443
Lactate	1.044 (1.012–1.077)	0.007

EEN, early enteral nutrition; BMI: body mass index; VDI, vasopressor dosing intensity; APACHE II score, Acute Physiology and Chronic Health Evaluation II score; SOFA score, sequential organ failure assessment score; HR, hazard ratio; CI, confidence interval.

**Figure 3:**
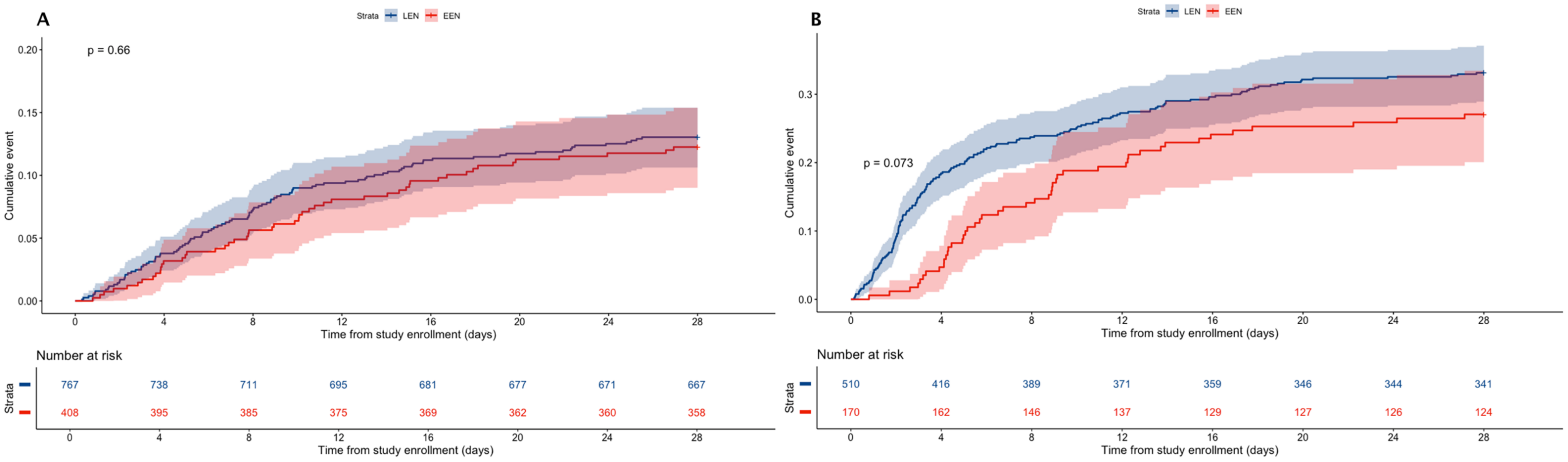
Kaplan–Meier curves illustrating cumulative incidence in septic patients, comparing the EEN and LEN groups, stratified by vasopressor dosing intensity (VDI) levels of < 15 μg/min **(A)** and ≥ 15 μg/min **(B)**. EEN, early enteral nutrition; LEN, late enteral nutrition; VDI, vasopressor dosing intensity.

Patients were stratified into two groups based on VDI: Low-VDI (<15 μg/min) and high-VDI (≥15 μg/min). In the low-VDI group, EEN exhibited a trend toward a reduced risk of 28-day mortality, although this difference failed to reach statistical significance [HR 0.738, 95% CI (0.523–0.1.042), *p* = 0.084; see Table 3; Figure 3A]. Conversely, in the high-VDI group, EEN had no discernible impact on 28-day mortality risk [HR 0.926, 95% CI (0.660–1.298), *p* = 0.655; see Table 3; Figure 3B].

**Table 3:** Association between EEN and 28-day mortality using the Cox proportional hazards regression model in patients with different VDI levels.

Variable	VDI < 15ug/min		VDI ≥ 15ug/min	
	HR (95% CI)	*p*-value	HR (95% CI)	*p*-value
EEN	0.738 (0.523–1.042)	0.084	0.926 (0.660–1.298)	0.655
Age	1.012 (1.000–1.024)	0.044	1.005 (0.997–1.014)	0.227
Male	1.012 (0.726–1.410)	0.946	1.061 (0.793–1.418)	0.691
BMI	0.948 (0.908–0.990)	0.015	0.974 (0.937–1.012)	0.171
VDI	1.017 (0.974–1.061)	0.452	1.007 (1.002–1.011)	0.004
APACHE II	1.074 (1.047–1.102)	<0.001	1.066 (1.044–1.089)	<0.001
SOFA score	1.035 (0.980–1.094)	0.216	0.993 (0.953–1.035)	0.739
Lactate	1.111 (1.038–1.188)	0.002	1.035 (1.001–1.071)	0.047

EEN, early enteral nutrition; VDI, vasopressor dosing intensity; BMI, body mass index; APACHE II score, Acute Physiology and Chronic Health Evaluation II score; SOFA score, sequential organ failure assessment score; HR, hazard ratio; CI, confidence interval.

Sensitivity analyses using other alternative VDI cutoffs in Cox proportional hazards regression models were performed. Although no statistically significant association was observed, EEN demonstrated a trend toward reduced mortality in patients with lower vasopressor requirements. Detailed results are presented in Supplementary Tables S2–S5.

## 4 Discussion

The present study, using the Chinese Database in Intensive Care (CDIC), suggests that EEN was not significantly associated with reduced 28-day mortality and that it only demonstrated a trend in septic patients requiring low-dose vasopressors, indicating that larger studies are needed to further explore the impact.

The findings of this study are consistent with those of previous studies. The NUTRIREA-2 study (19) found that EEN did not significantly reduce mortality or secondary infections, but it was linked to an increased risk of digestive complications in patients receiving high-dose norepinephrine. The discrepancy may be attributed to differences in illness severity and the volume of enteral nutrition administered. In the present study, the SOFA score was 11, consistent with the NUTRIREA study, indicating a comparable severity of illness between the study populations. The administered dosage of vasopressors was also relatively lower in our study (0.15ug/kg/min) compared to that reported in the NUTRIREA-2 study (0.56ug/kg/min).

EEN may be beneficial for septic patients whose hemodynamics are “relatively stable,” defined as requiring lower doses of vasopressors. The results of this study are consistent with those reported by Ohbe et al. (20), suggesting a similar trend or outcome. In this study, the 28-day mortality rate was reduced and the prognosis was favorable for patients with VDI < 15ug/min, which may be associated with better gastrointestinal tolerance. In a retrospective study of 319 patients receiving enteral nutrition with (highest norepinephrine dose of 0.16 ug/ kg/min) or without vasopressor support, Sabino et al. (21) found no differences in the rates of bowel ischemia or emesis between groups. Among the four reported cases of mesenteric ischemia, two occurred in patients not receiving vasopressors. In addition, Qi et al. (22) recently found that patients with septic shock who tolerated enteral nutrition had lower norepinephrine doses [0.23 vs. 0.28 ug/kg/min, *p* = 0.049]. In septic patients, the initiation of EEN is associated with improved outcomes, potentially due to better gastrointestinal tolerance resulting from the administration of low-dose vasopressors.

Enteral nutrition in patients receiving higher norepinephrine doses is not associated with a reduction in 28-day mortality. In a propensity-matched analysis, EEN was not associated with a reduction in mortality in patients requiring high-dose noradrenaline (>0.3ug/kg/min) (20). In the present study, we also observed that EEN was not associated with improved mortality in the high-VDI group. Higher vasopressors may promote Enteral Feeding Intolerance (EFI) and gastrointestinal dysfunction. A prospective observational study (23) showed that in critically ill patients receiving enteral nutrition while requiring vasoactive drugs, the mean dose of norepinephrine was 0.71 mcg/kg/min during the first 48 h, and 77% patients experienced EN-related complications. The NUTRIREA-2 and NUTRIREA-3 studies reported baseline norepinephrine doses of at least 0.5ug/kg/min, which were higher than those in other contemporary studies evaluating the use of enteral nutrition in shock (19, 24). This may promote EFI. In an observational study, Wang et al. (25) divided 66 patients with circulatory shock receiving enteral nutrition (more than 80% with septic shock) into those who developed EFI and those who did not. They found that a mean NEE dose of 0.2 ug/kg/min predicted EFI with a sensitivity of 88%. EEN may not be recommended for patients with unstable hemodynamic status requiring high-dose norepinephrine.

In the present study, we defined a low dose of norepinephrine as VDI<15 μg/min, according to previous studies (26, 27). The VASST (27) defined the stratum of less severe septic shock as treatment with 5 to 14 μg of norepinephrine (or equivalent) per minute, and the stratum of more severe septic shock was defined as treatment with 15 μg or more of norepinephrine (or equivalent) per minute. In addition, the VOLUME-CHASERS analysis (26) also used the same cut-off point for a low dose (VDI < 15 μg/min NEE).

Should we delay EN in patients with shock receiving vasopressors or inotropes? Current clinical guidelines do not recommend EEN in patients with unstable or uncontrolled hemodynamics and suggest delaying EN until hemodynamics are controlled, while initiating low-dose enteral nutrition as soon as possible thereafter. To date, no RCTs have been identified (9), and more evidence is needed to confirm its benefits. Our study demonstrates that EEN does not correlate with reduced mortality in patients receiving high-dose vasopressors (VDI ≥ 15ug/min). Therefore, a norepinephrine (or equivalent) dose of ≥15ug/min may represent a plausible cutoff point above which EEN is not indicated.

Interestingly, EEN patients had longer ICU and hospital length of stay (LOS), despite a trend toward lower mortality. This may be explained by the following: (1) a competing risk effect, whereby higher mortality in the LEN group (21.1% vs. 16.6%) led to shorter LOS due to earlier deaths; (2) baseline differences, as EEN patients were older and had greater illness severity, more frequent pneumonia, and higher IMV use, all of which are associated with prolonged recovery; and (3) a survival effect, whereby EEN may have enabled critically ill patients to survive longer, thereby accruing more hospital days. These factors should be considered when interpreting LOS outcomes.

There are also several limitations of this study that warrant acknowledgement. First, this is a single-center, retrospective cohort study that establishes the association but does not confirm causality. Consequently, well-designed randomized controlled trials (RCTs) are needed to validate these findings. Second, our focus was on the impact of the timing of enteral nutrition on mortality, without considering total caloric and protein intake, which are also critical components of nutrition support and may significantly influence patient outcomes. Third, the allocation of EEN was not randomized, as this was a real-world observational study; therefore, the results might be influenced by several confounders. In addition, the sample size of patients receiving EEN might have been insufficient. Finally, although the vasopressor dosing intensity (VDI) provides a method to quantify the intensity of vasopressor therapy, it may not accurately represent individual pharmacological equivalence. Nonetheless, this metric, consistent with previous research, offers a standardized approach for comparing the dosages of different vasopressor agents.

## 5 Conclusion

This study indicates that EEN is not associated with a reduction in mortality rates among critically ill septic patients requiring vasopressors, although a trend was observed in those receiving low-dose vasopressors (VDI<15ug/min). No significant difference was found in patients with unstable hemodynamics (VDI ≥ 15ug/min), and larger studies are needed to explore the impact.

## Data Availability

The raw data supporting the conclusions of this article will be made available by the authors, without undue reservation.
