# Supplementary material for: The impact of early enteral nutrition on 28-day mortality in septic shock: a cohort study
Source: Front Nutr. 2026 May 11;13:1789277. doi: 10.3389/fnut.2026.1789277 (PMC13199022; doi:10.3389/fnut.2026.1789277)
Supplement: Supplementary file 1 [file Table_1.docx]

Supplementary Material

**Additional Methods:**

**A. Data Collection**

Comorbidities were extracted from the CDIC database including congestive heart failure (CHF), atrial fibrillation (AFIB), chronic renal disease, liver disease, chronic obstructive pulmonary disease (COPD), stroke, and malignant tumor and identified on the basis of the recorded ICD-10 codes. Initial lactate levels and vital signs, including mean arterial pressure (MAP), heart rate, temperature (°C), and respiratory rate, were also extracted.

**B. Vasopressor Dosing Intensity (VDI)**

VDI was quantified as the total vasopressor dose infused across all vasopressors in NEE and expressed in ug/min [1] [2]. We chose ug/min (as opposed to ug/kg/min) because most of the studied used ug/min for norepinephrine dosing. VDI was determined by first taking the average of the lowest and highest infusion dose documented for each vasopressor at each time period, and then calculating the corresponding NEE for that time period for each vasopressor administered. This calculation assumes that the patient was maintained at a constant infusion dose midway between the lowest and highest infusion doses over each time period. Once VDI was obtained for each time period (hour 0–3, 3–6, 6–12, and 12–24), we calculated the cumulative average VDI at 24 hours by taking a time-weighted average of VDI from 0–24 hours. In a secondary analysis that aimed to examine the timing of vasopressor titration, we categorized VDI for the time period of 0–24 hours as low- dose (VDI < 15μg/min NEE) or high-dose (VDI ≥ 15μg/min NEE) [2] [3] [4] [5]. Vasopressor requirement as norepinephrine equivalent dose was calculated as [norepinephrine (ug/min)] + [dopamine (ug/kg/min) ÷ 2] + [epinephrine (ug/min)] + [phenylephrine (ug/min) ÷ 10] after the study of Russell [2]introduced in the additional paper and the review which showed the conversion of different vasopressor dose in the calculation.

**Reference**

[1] Qi F, Huang G, Li H, Zhao X, Liu J. Correlation analysis of norepinephrine dose on enteral nutrition tolerance and prognosis in patients with septic shock. BMC Infect Dis 2023;23(1):386. doi: 10.1186/s12879-023-08366-x.

[2] Russell JA, Walley KR, Singer J, Gordon AC, Hebert PC, Cooper DJ, et al. Vasopressin versus norepinephrine infusion in patients with septic shock. N Engl J Med 2008;358(9):877-87. doi: 10.1056/NEJMoa067373.

[3] Sabino KM, Fuller J, May S, Wakefield D. Safety and Tolerance of Enteral Nutrition in the Medical and Surgical Intensive Care Unit Patient Receiving Vasopressors. Nutr Clin Pract 2021;36(1):192-200. doi: 10.1002/ncp.10548.

[4] Flordelis Lasierra JL, Montejo Gonzalez JC, Lopez Delgado JC, Zarate Chug P, Martinez Lozano-Aranaga F, Lorencio Cardenas C, et al. Enteral nutrition in critically ill patients under vasoactive drug therapy: The NUTRIVAD study. JPEN J Parenter Enteral Nutr 2022;46(6):1420-30. doi: 10.1002/jpen.2371.

[5] Roberts RJ, Miano TA, Hammond DA, Patel GP, Chen JT, Phillips KM, et al. Evaluation of Vasopressor Exposure and Mortality in Patients With Septic Shock. Crit Care Med 2020;48(10):1445-53. doi: 10.1097/CCM.0000000000004476.

**Table S1: Baseline characteristics of cohort trials**

|  | Total (n = 1184) | EEN (n = 354) | LEN (n = 830) | p value |
| --- | --- | --- | --- | --- |
| Height, cm | 170 (160, 172) | 170 (160, 173) | 168 (160, 172) | 0.041 |
| Weight, kg | 65 (60, 70) | 65 (60, 70) | 65 (60, 70) | 0.506 |
| GCS | 11 (6, 15) | 10 (6, 15) | 13 (6, 15) | < 0.001 |
| Na+, mmol/L | 139 (135, 142) | 139 (135, 142) | 139 (135, 142) | 0.937 |
| K+, mmol/L | 3.7 (3.3, 4.1) | 3.6 (3.3, 4.2) | 3.7 (3.3, 4.1) | 0.807 |
| Hct, % | 31 (25.9, 36.3) | 31 (26.9, 37) | 30.8 (25.4, 36) | 0.106 |
| WBC, 10^9/L | 11.9 (8.4, 17.1) | 11.6 (7.9, 16.6) | 12.1 (8.4, 17.3) | 0.22 |
| UO, ml/24 hours | 2648 (1674, 3651) | 2620 (1696, 3601) | 2685 (1659, 3734) | 0.771 |
| IL-6, mmol/L | 157 (57, 486) | 118 (45, 420) | 184 (62, 582) | 0.016 |
| Prealbumin, g/L | 0.13 (0.09, 0.18) | 0.13 (0.08, 0.18) | 0.14 (0.1, 0.19) | 0.097 |
| Albumin, g/L | 30 (26, 34) | 31 (27, 34) | 29 (25, 34) | < 0.001 |

GCS, Glasgow Coma Scale; WBC, White Blood Cell; UO, Urinary output; IL -6, Interleukin 6

Table S2: The association between EEN and 28-day mortality using Cox proportional-hazards regression model in patients with different VDI.

|  | VDI<10ug/min | | VDI≥10ug/min | |
| --- | --- | --- | --- | --- |
|  | HR (95% CI) | *P* Value | HR (95% CI) | *P* Value |
| EEN | 0.749 (0.502–1.117) | 0.156 | 0.863 (0.637–1.170) | 0.342 |
| Age | 1.008 (0.996–1.021) | 0.206 | 1.007 (0.999–1.015) | 0.086 |
| Male | 0.990 (0.674–1.455) | 0.961 | 1.090 (0.836–1.421) | 0.524 |
| BMI | 0.926 (0.881–0.973) | 0.003 | 0.983 (0.950–1.017) | 0.314 |
| VDI | 1.037 (0.962–1.118) | 0.342 | 1.008 (1.004–1.012) | <0.001 |
| APACHE II | 1.080 (1.049–1.112) | <0.001 | 1.068 (1.047–1.088) | <0.001 |
| SOFA score | 1.040 (0.976–1.107) | 0.225 | 0.996 (0.958–1.035) | 0.828 |
| Lactate | 1.100 (1.005–1.205) | 0.040 | 1.038 (1.004–1.074) | 0.028 |

EEN, early enteral nutrition; VDI, vasopressor dosing intensity; BMI: body mass index; APACHE II score, acute physiology and chronic health evaluation II score; SOFA, sepsis-related organ failure assessment) score; HR, hazard ratio; CI, confidence interval

Table S3: The association between EEN and 28-day mortality using Cox proportional-hazards regression model in patients with different VDI.

|  | VDI<14ug/min | | VDI≥14ug/min | |
| --- | --- | --- | --- | --- |
|  | HR (95% CI) | *P* Value | HR (95% CI) | *P* Value |
| EEN | 0.708 (0.498–1.007) | 0.055 | 0.942 (0.678–1.311) | 0.724 |
| Age | 1.012 (1.001–1.025) | 0.041 | 1.005 (0.997–1.014) | 0.220 |
| Male | 1.047 (0.745–1.470) | 0.792 | 1.050 (0.789–1.397) | 0.738 |
| BMI | 0.939 (0.899–0.981) | 0.005 | 0.981 (0.945–1.019) | 0.320 |
| VDI | 1.025 (0.978–1.074) | 0.304 | 1.007 (1.003–1.012) | 0.002 |
| APACHE II | 1.074 (1.047–1.102) | <0.001 | 1.068 (1.046–1.090) | <0.001 |
| SOFA score | 1.038 (0.982–1.097) | 0.189 | 0.993 (0.953–1.034) | 0.735 |
| Lactate | 1.100 (1.017–1.189) | 0.017 | 1.037 (1.002–1.073) | 0.038 |

EEN, early enteral nutrition; VDI, vasopressor dosing intensity; BMI: body mass index; APACHE II score, acute physiology and chronic health evaluation II score; SOFA, sepsis-related organ failure assessment) score; HR, hazard ratio; CI, confidence interval

Table S4: The association between EEN and 28-day mortality using Cox proportional-hazards regression model in patients with different VDI.

|  | VDI<18ug/min | | VDI≥18ug/min | |
| --- | --- | --- | --- | --- |
|  | HR (95% CI) | *P* Value | HR (95% CI) | *P* Value |
| EEN | 0.762 (0.551–1.053) | 0.099 | 0.921 (0.642–1.320) | 0.653 |
| Age | 1.013 (1.002–1.024) | 0.019 | 1.004 (0.995–1.013) | 0.429 |
| Male | 0.979 (0.714–1.343) | 0.894 | 1.115 (0.825–1.507) | 0.479 |
| BMI | 0.951 (0.913–0.991) | 0.017 | 0.971 (0.934–1.011) | 0.150 |
| VDI | 1.016 (0.981–1.051) | 0.372 | 1.006 (1.002–1.011) | 0.010 |
| APACHE II | 1.075 (1.049–1.101) | <0.001 | 1.065 (1.043–1.089) | <0.001 |
| SOFA score | 1.037 (0.985–1.092) | 0.171 | 0.990 (0.949–1.033) | 0.635 |
| Lactate | 1.094 (1.018–1.176) | 0.015 | 1.036 (1.001–1.072) | 0.046 |

EEN, early enteral nutrition; VDI, vasopressor dosing intensity; BMI: body mass index; APACHE II score, acute physiology and chronic health evaluation II score; SOFA, sepsis-related organ failure assessment) score; HR, hazard ratio; CI, confidence interval

Table S5: The association between EEN and 28-day mortality using Cox proportional-hazards regression model in patients with different VDI.

|  | VDI<20ug/min | | VDI≥20ug/min | |
| --- | --- | --- | --- | --- |
|  | HR (95% CI) | *P* Value | HR (95% CI) | *P* Value |
| EEN | 0.785 (0.576–1.072) | 0.128 | 0.907 (0.618–1.331) | 0.618 |
| Age | 1.013 (1.002–1.023) | 0.017 | 1.004 (0.994–1.013) | 0.457 |
| Male | 1.061 (0.781–1.442) | 0.705 | 1.028 (0.754–1.402) | 0.860 |
| BMI | 0.949 (0.912–0.988) | 0.011 | 0.974 (0.935–1.015) | 0.213 |
| VDI | 1.021 (0.992–1.052) | 0.162 | 1.006 (1.001–1.011) | 0.014 |
| APACHE II | 1.073 (1.048–1.099) | <0.001 | 1.067 (1.043–1.092) | <0.001 |
| SOFA score | 1.021 (0.971–1.074) | 0.420 | 0.998 (0.955–1.043) | 0.916 |
| Lactate | 1.120 (1.050–1.195) | <0.001 | 1.028 (0.992–1.065) | 0.129 |

EEN, early enteral nutrition; VDI, vasopressor dosing intensity; BMI: body mass index; APACHE II score, acute physiology and chronic health evaluation II score; SOFA, sepsis-related organ failure assessment) score; HR, hazard ratio; CI, confidence interval

Table S6: The association between EEN and 28-day mortality using Cox proportional-hazards regression model in patients with different EN Volume at 24hours.

|  | EN volume<500mL | | EN volume≥500mL | |
| --- | --- | --- | --- | --- |
|  | HR (95% CI) | *P* Value | HR (95% CI) | *P* Value |
| EEN | 0.819 (0.490–1.369) | 0.446 | 0.805 (0.522–1.242) | 0.327 |
| Age | 1.005 (0.997–1.013) | 0.194 | 1.017 (1.002–1.031) | 0.027 |
| Male | 0.968 (0.753–1.245) | 0.801 | 1.350 (0.852–2.139) | 0.201 |
| BMI | 0.963 (0.930–0.997) | 0.031 | 0.956 (0.908–1.007) | 0.092 |
| VDI | 1.006 (1.002–1.011) | 0.003 | 1.020 (1.007–1.033) | 0.002 |
| APACHE II | 1.083 (1.063–1.103) | <0.001 | 1.052 (1.017–1.087) | 0.003 |
| SOFA score | 1.009 (0.972–1.048) | 0.630 | 1.024 (0.955–1.097) | 0.508 |
| Lactate | 1.037 (1.002–1.072) | 0.036 | 1.085 (0.982–1.199) | 0.108 |

EEN, early enteral nutrition; VDI, vasopressor dosing intensity; BMI: body mass index; APACHE II score, acute physiology and chronic health evaluation II score; SOFA, sepsis-related organ failure assessment) score; HR, hazard ratio; CI, confidence interval
